# Personal meaning of work and perceived work ability among middle-aged workers with physically strenuous work: a Northern Finland Birth Cohort 1966 Study

**DOI:** 10.1007/s00420-019-01412-9

**Published:** 2019-02-14

**Authors:** Anne Punakallio, Sirpa Lusa, Leena Ala-Mursula, Ellen Ek, Nina Nevanperä, Jouko Remes, Juha Auvinen, Jorma Seitsamo, Jaro Karppinen, Jaana Laitinen

**Affiliations:** 1 Finnish Institute of Occupational Health, PO Box 40, 00032 Työterveyslaitos, Helsinki, Finland; 2 Center for Life Course Health Research, University of Oulu, Oulu, Finland; 3 Medical Research Center Oulu, Oulu University Hospital and University of Oulu, Oulu, Finland

**Keywords:** Birth cohort, Work ability, Personal meaning of work, Physically strenuous work, Longitudinal study

## Abstract

**Purpose:**

To investigate the association between personal meaning of work and perceived work ability among middle-aged workers with physically strenuous or light work. We evaluated the course of perceived work ability from 31 to 46 years and examined the possible differences in the association between personal meaning of work and perceived work ability at the age of 46 depending on physical workload.

**Methods:**

The study population consisted of participants of the Northern Finland Birth Cohort 1966 (*n* = 4420). Data were collected through questionnaires at 31 and 46 years. The main outcome was perceived work ability (0–7 = poor, 8–10 = good) and the main explanatory measures were physically strenuous work and personal meaning of work. Multivariate logistic regression analyses were adjusted for unhealthy habits, number of diseases, job strain, social support at work, employment history and gender. They were also stratified for the strenuousness of work.

**Results:**

Perceived work ability decreased during the 15-year follow-up in both the strenuous and light work groups, and was lowest among workers with strenuous work. Perceived work ability remained poor or decreased in 22% of men and 21% of women in the strenuous work group vs. 14% and 13% in the light work group, respectively. After adjusting for confounders, the participants in both groups who reported low personal meaning of work were at approximately a twofold risk of having poor perceived work ability at 46 years compared to the participants who reported high personal meaning of work.

**Conclusions:**

Perceived work ability was significantly lower and deteriorated more during the follow-up among participants with strenuous work. High personal meaning of work was important for good work ability, irrespective of the strenuousness of work.

## Introduction

The need to extend working careers is serious in western societies, due to the changed age structure of the populations (Eurostat, New Cronos, http://ec.europa.eu/eurostat/data/database). Work ability is an important determinant of the length of working careers, and poor work ability in midlife predicts disability severity years later (von Bonsdorff et al. 2012), sickness absences (Sell et al. 2009) and early retirement (Roelen et al. 2014). Maintaining the work ability of employees is an important prerequisite for preventing early exit from work and for increasing productivity (Ilmarinen 2006).

**Fig. 1:**
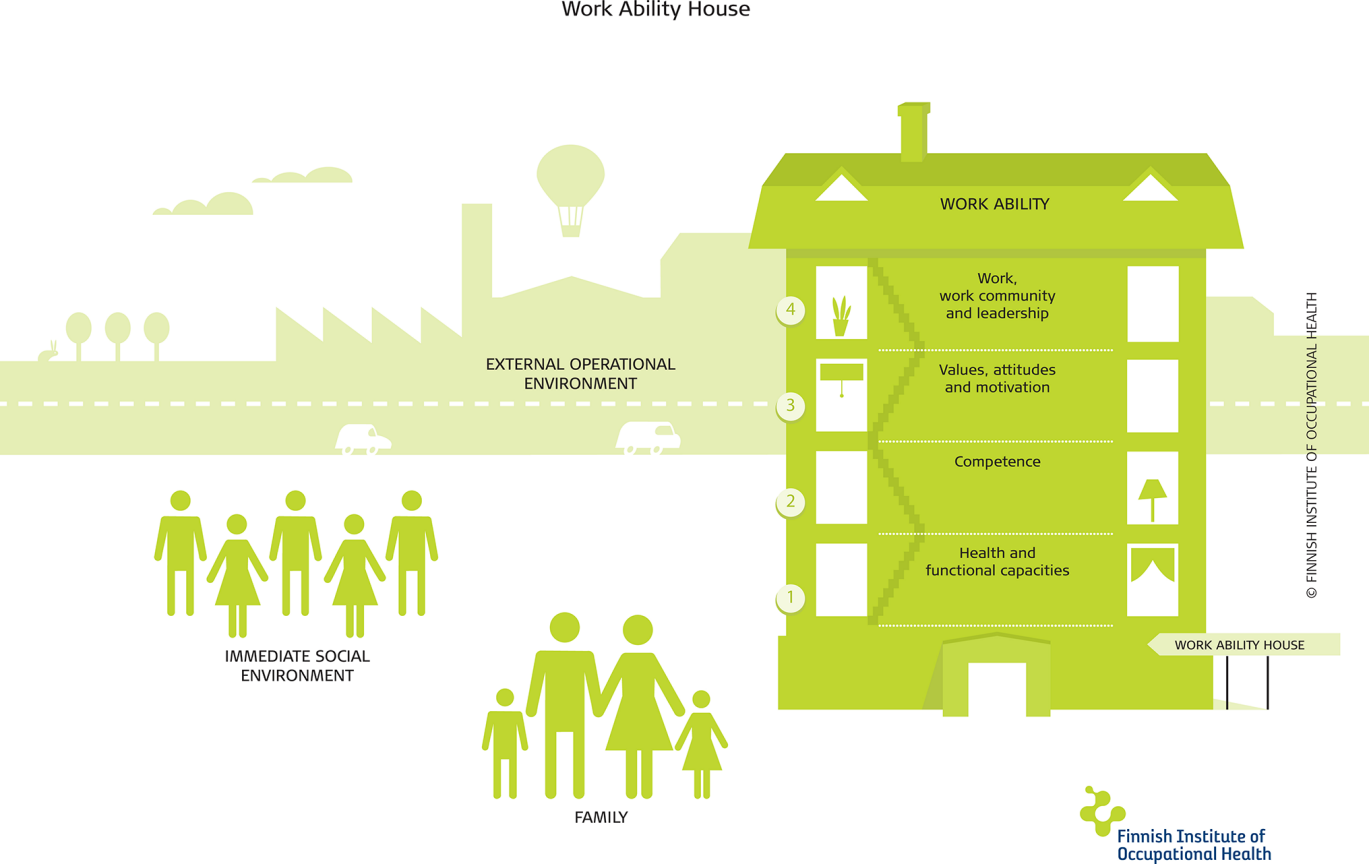
The Work Ability House (Ilmarinen et al. 2005)

The concept of work ability has many complex, holistic and dynamic aspects (for a scoping review, see Lederer et al. 2014). One way of conceptualizing and visualizing it is the “Work Ability House”, which has four floors indicating four dimensions of work ability that interact with each other (Ilmarinen et al. 2005) (Fig. 1). In this conceptualization, health, health habits, physical and mental capacities form the basic floor. The other floors are comprised of occupational competence; of personal aspects such as values, attitudes and motivation; and last, of work itself, including the content and demands of work, the work environment, the community and the organization. In addition, external circumstances related to family, the social environment and the (macroeconomic) circumstances in the economy and society influence a worker’s work ability.

Empirically, studies have shown that the ground floor of health and functional capacities, and the floor of work-related factors have the strongest effect on work ability, especially among older workers (Gould et al. 2008; Ilmarinen et al. 2005). In general, the floor consisting of values, attitudes and motivational factors, including personal meaning of work, has not received much attention in the research on work ability. The personal sense of meaning in any domain is, however, fundamental in all human intentional action (Frankl 1963; O’Connor and Chamberlain 1996). According to Kahn and Wiener (1967), work can fulfil the need for short- or long-term income, and at the same time it can satisfy internal values such as exercising and mastering gratifying skills, participating in an important activity, self-identification and self-fulfilment. Kahn and Wiener also propose that there are individual differences in how these work-related gains are perceived and valued, resulting in differing levels of personal meaning of work in an individuals’ life.

In terms of opportunities to support work ability, the floor of work, including physical and psychosocial work environment and physical work demands, entitles a natural platform for work-related actions, and advancing technologies have indeed been helpful in the overall reduction of physical work demands during the last decades. Still, it is not always possible to avoid excessive physical workload or demanding postures. Even to date, work demands may remain physically hard. Early retirement due to loss of work ability is most common in physically demanding occupations (Pensola et al. 2010). Among middle-aged workers (mean 47, range 44–51 years), work ability decreased more in physical work than in mental work over an 11-year follow-up (Ilmarinen et al. 1997). The decrease was more significant among the oldest workers (over 51 years), the highest in physically strenuous work and the lowest in mentally strenuous work (Ilmarinen et al. 1997). However, whether the early development of work ability up to midlife differs between those with and without physically strenuous work is not as well known.

The increased risk of work disability among those with physically strenuous work can be due to many reasons. It may relate to selection to physically strenuous work, to educational or lifestyle factors, or to high physical strain itself (Punakallio et al. 2014; von Bonsdorff et al. 2011). A combination of obesity-related diseases and physically strenuous work has been shown to have a cumulative deleterious effect on work ability (Gould et al. 2008). Lack of vigorous physical activity in leisure time, obesity, psychosocial work-related factors such as high mental work demands, lack of autonomy, poor task resources, poor leadership and relationships between workers and supervisors, as well as lack of social support, poor physical work environment, and high physical workload have all been associated with poor work ability (Airila et al. 2012; Lusa et al. 2011; Nevanperä et al. 2015, 2016; van den Berg et al. 2009). In addition, poor musculoskeletal capacity, motor coordination and balance, and work accidents and poor working postures may lead to decreased work ability among workers in physically demanding occupations (Lusa et al. 2011; Punakallio et al. 2011).

A recent study observed that the task-based work engagement was positively associated with work ability even after adjusting for age, life habits and working conditions among workers with a heavy physical workload (Airila et al. 2012). Analogously, low perceived meaning at work, operationalized as a feeling that work is not important, not meaningful, and not feeling motivated and engaged in one’s work, was associated with earlier disability pensioning (Clausen et al. 2014). Instead, less is known of the relevance of more personal meaning of work for work ability in physically strenuous work. To our knowledge, no previous longitudinal study has explored whether the associations between personal meaning of work and work ability differ among workers doing physically strenuous or light work.

The goal of this study was to examine the association between the personal meaning of work and perceived work ability among middle-aged participants with and without physically strenuous work. The specific aims were: (1) to evaluate the development of work ability from 31 to 46 years in physically strenuous compared to light work and (2) to describe any differences in the association between the personal meaning of work and work ability among workers with and without physically strenuous work.

## Methods

### Study population and data collection

**Fig. 2:**
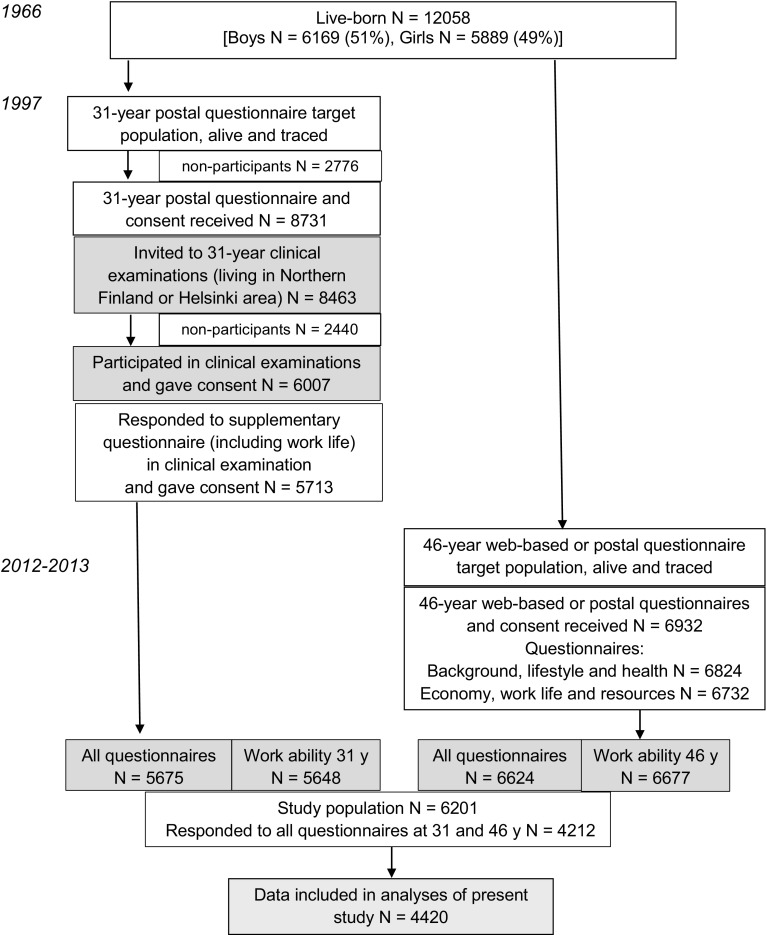
Flowchart of the Northern Finland Birth Cohort 1966 (Rantakallio 1969, 1988)

The ongoing Northern Finland Birth Cohort (NFBC) 1966 started with a study population comprising 96.3% of all births during 1966 in the areas of Oulu and Lapland, and was followed up for 46 years (Rantakallio 1969, 1988) (Fig. 2).

In the 31-year follow-up in 1997, a postal questionnaire was sent to participants who were alive and had a known address (*n* = 11,541), and 75.3% responded (Järvelin et al. 2004). Cohort members who lived in Northern Finland or in the metropolitan area (*n* = 8463) were invited to a clinical examination, during which they were asked to reply to a questionnaire about work life. Of these, 67.5% (*n* = 5713) responded. In the 46-year follow-up, 10,321 participants were alive and traced, and asked by letter to fill in web-based questionnaires. If the participants had no computer or preferred answering on paper, they were sent a postal questionnaire. Answers were received from 67.4% (*n* = 6932) of all invited participants.

The final study population included those participants whose data on the studied variables at 31 and 46 years were available (*n* = 4420) (Fig. 2). The effect of potential selection bias was studied by comparing the distribution of some variables of those included in the results to those excluded from the analyses due to missing data.

### Compliance with ethical standards

All participants gave a written informed consent in accordance with the Declaration of Helsinki 1975, as revised in 2000, at each stage of the study. The study was approved by the Ethics Committee of the Northern Ostrobothnia Hospital District.

### Outcome measure

*Current perceived work ability* compared to lifetime best was used as the outcome measure. The participants evaluated their current perceived work ability on a scale of 0–10 at the ages of 31 and 46, 10 indicating lifetime best work ability. The question used for this was the first item of the Work Ability Index (Tuomi et al. 1991, 1997, 2006). Current perceived work ability is a reliable and valid indicator of work ability (de Zwart et al. 2002; van den Berg et al. 2009; Tuomi et al. 1998). It has associated strongly with the whole Work Ability Index and predicted mental and physical work strain in midlife and disability after retirement (Ahlström et al. 2010; el Fassi et al. 2013; Ilmarinen and Tuomi 2004; von Bonsdorff et al. 2011, 2012).

Current perceived work ability was first classified into two groups: good (8‒10) and poor (0‒7). Second, to describe perceived work ability from 31 to 46 years, the item was divided into four groups: always good (8‒10), deteriorated (from 8‒10 to 0‒7), improved (from 0‒7 to 8‒10), and always poor (0‒7).

### Explanatory measures

The main explanatory measures were physically strenuous work and personal meaning of work. Physically strenuous work was evaluated at the age of 31 and 46, using the question “To what extent are the following tasks and postures part of your job?” The participants had to evaluate certain tasks and postures in their work, through nine items: “Heavy physical work in which the body has to struggle”, “Lifting loads of 1 to 15 kg”, “Lifting loads over 15 kg”, “Continuous movement or walking”, “Repetitious work movements”, “Standing”, “Working with the upper arms elevated”, “Forward-bent work postures” and “Rotational movements of the trunk”. The response scale was from 1 to 5; 1 (not at all or very rarely), 2 (rarely), 3 (moderately), 4 (often), and 5 (very often). The scale was reclassified as physically light work (light work, 1–2) and strenuous work (strenuous work, 3–5). We summed the recoded answers of nine questions and divided the scores into two groups, using the median as cut-offs.

Personal meaning of work was evaluated at the age of 46 using the scale introduced by Kahn and Wiener (1967), (Ek et al. 2005) by asking “How well do the following statements apply to you? with six items: “Work is an unpleasant necessity for earning money”; “Work brings you satisfaction, because you learn to use different occupational skills”; “Work is important, because it gives you experiences of accomplishment and progress”; “Work is a calling that allows you to fulfil yourself”; and “Work is the purpose of life and allows you to accomplish significant things”. The response scale was from 1 to 5; 1 (very little), 2 (little), 3 (moderately), 4 (much) and 5 (very much) and the first item was used as reversed. Cronbach’s alpha for the scale was 0.798. Next, we recoded the values 1 = 0, 2 = 1, 3 = 2, 4 = 3, 5 = 4 and calculated the sum variable using the recoded values. Finally, the sum scores were divided into three groups, using the tertiles as cut-offs; 1 = 0–10 (low), 2 = 11–14 (medium), 3 = ≥ 15 (high).

### Covariates

Multivariate analyses were adjusted for job strain and social support at work, representing the floor of work in the Work Ability House, for self-reported employment history indicating the participant’s attachment to work, as well as for number of diseases and health habits in reflection of the basic floor of health in the Work Ability House. The selection of poor health habits (leisure time physical inactivity, smoking, alcohol consumption, stress-related eating and drinking) was based on the results of an earlier Finnish population-based study by Laaksonen et al. (2001). The data on all these covariates were obtained from questionnaires at the age of 46.

*Job strain* psychosocial job characteristics (i.e. job demands and job control) were evaluated using questions from the Job Content Questionnaire (Karasek et al. 1998). Job demands (11 items) and job control (15) were evaluated on a scale of 1 (very little) to 5 (very much), as previously reported (Kujala et al. 2006). The scores of both characteristics were summed and divided into two groups (high/low), based on median splits. Four further groups were created; high demands and high control (active), high demands and low control (high strain), low demands and high control (low strain), and low demands and low control (passive) (Karasek et al. 1998). Cronbach’s alpha values for scales of job control and job demands were 0.88 and 0.90, respectively.

The amount of social support at work was evaluated using a structured five-point scale that elicited the extent (a lot—not at all) to which the participant received emotional support (listening or advice) or practical support (help with a work task) from coworkers and supervisors (four items). The sum of the four items was calculated and recoded as (1) little or no support, (2) some support, and (3) a great deal of support (Kujala et al. 2006). Cronbach’s alpha for the scale was 0.84.

*Employment history* was elicited by the question: which of the following seven alternatives (from always continuously employed to never in gainful employment) best describes your employment history? We divided the scores into two groups, using the medians as cut-offs (continuously employed vs. at least temporary unemployed).

*Number of diseases* was the sum of self-reported physician-diagnosed diseases elicited using the Work Ability Index (Kujala et al. 2005; Tuomi et al. 2006).

*Physical activity* was evaluated by eliciting participation in light and brisk leisure time physical activity/exercise. Physical activity was classified into three groups: inactive (brisk physical activity less than once a week and light activity less than four times a week), moderately active (brisk physical activity at least once a week but less than 20 min at a time or light physical activity at least four times a week) and active/very active (brisk physical activity at least two times a week, at least 20 min at a time) (Tammelin et al. 2003).

*Smoking* was classified as ex-smoker/never smoked, light smoker (5–6 days a week or occasionally) or smoker (daily smoking).

*Alcohol consumption* was evaluated on the basis of the frequency of alcohol use (daily to once a year or never) and the usual amount of each alcoholic beverage [beer/cider/long drink (a Finnish beverage, equal in strength to beer and cider), light wine, table wine and spirits] per drinking occasion (Nevanperä et al. 2016). From these, we calculated the weekly consumption (portions/week) and formed three groups on the basis of the tertile cut-offs. The cut-off points were < 1.5, ≥ 1.5 to < 10 and ≥ 10 for men, and < 1, ≥ 1 to < 5 and ≥ 5 portions/week for women.

*Stress-related eating and drinking* is an indicator of a passive coping style and is associated with poor perceived work ability (Nevanperä et al. 2015). This was measured by asking the participants to evaluate if they had tried to relieve feelings of stress by eating, drinking, using medication, etc. the last time they had felt stressed (Folkman and Lazarus 1985; Lazarus and Folkman 1984). This one item of the Ways of Coping Checklist has been used in earlier studies among adolescents and adults (Jääskeläinen et al. 2014; Laitinen et al. 2002). The answers were classified into two groups; no = 0 (not at all, somewhat) and yes = 1 (quite a lot or a great deal) (Laitinen et al. 2002). The sum score including stress-related eating was calculated (and divided into two groups; no (sum score of 0) and yes (sum score of 1–2).

*Health habits* included physical inactivity, current smoking, risky alcohol consumption, and stress-related eating and drinking. At the age of 46, they were further combined and three groups (healthy, between and unhealthy) were formed on the basis of the tertiles of the sum scores (physical inactivity, smoking, alcohol consumption, stress-related eating and drinking).

### Statistical analyses

The statistical analyses were performed using IBM SPSS Statistics 23 for Windows (IBM Corp., Armonk, NY, USA). The course of perceived work ability (0–10) from 31 to 46 years among men and women according to the physical strenuousness of the work were described. The differences between the median perceived work ability values of the subgroups were investigated by reporting 95% confidence intervals (CI) and using the Mann–Whitney-*U* test.

We used cross-tabulation and chi-square tests to investigate the univariate associations between explanatory variables and perceived work ability at the age 46. Multivariate logistic regression analyses were used to calculate risk ratios (RR) and their 95% confidence intervals (CI) for poor perceived work ability at 46 years. Personal meaning of work was used as an explanatory variable, and models were adjusted for gender, job strain, social support at work, employment history, health habits, and number of diseases, all at the age of 46. The analyses were stratified by physical strenuousness of work.

## Results

### Work ability from 31 to 46 years

Perceived work ability at the age of 31 was lower among workers with physically strenuous work than among those with physically light work, among both men and women (Table 1). Perceived work ability decreased during the 15-year follow-up in both groups, and was the lowest among workers with strenuous work. At the age of 46, 22% of men and 20% of women had poor perceived work ability in the strenuous work group, and 14% of men and 13% of women had poor perceived work ability in the light work group, respectively. In the strenuous work group, perceived work ability remained always poor or decreased among 22% of men and 21% of women in comparison with 14% and 13%, respectively, in the light work group (Table 1).

**Table 1:** Course of perceived work ability (PWA) (0–10) from 31 to 46 years among men and women according to physical strenuousness of work

Physically strenuous work at 31 years						
	Men			Women		
	Yes	No	*p*	Yes	No	*p*
PWA, mean (95% CI)						
31 years	8.7 (8.7–8.8)	9.0 (9.0–9.1)		8.7 (8.7–8.8)	9.0 (8.9–9.1)	
	(*n* = 1107)	(*n* =1088)		(*n* = 1058)	(*n* = 970)	
46 years	8.1 (8.0–8.2)	8.6 (8.5–8.7)		8.2 (8.1–8.4)	8.6 (8.5–8.7)	
	(*n* = 790)	(*n* = 824)		(*n* = 852)	(*n* = 790)	
Poor PWA, *n* (%)						
31 years	120 (11)	82 (8)		123 (12)	65 (7)	
			0.007			< 0.001
46 years	174 (22)	112 (14)		174 (20)	99 (13)	
PWA, *n* (%)			< 0.001			< 0.001
Always poor	32 (4)	23 (3)		40 (5)	16 (2)	
Deteriorated	140 (18)	87 (11)		132 (16)	83 (11)	
Improved	49 (6)	34 (4)		53 (6)	33 (4)	
Always good	557 (72)	670 (82)		618 (73)	654 (83)	
			< 0.001			< 0.001

The participants whose work remained strenuous during the whole follow-up period had the worst perceived work ability; men 8.3 (8.2–8.4) and women 8.4 (8.2–8.5). In this group, 23% of men and 19% of women remained under the perceived work ability score of 8, whereas in the group in which the work remained light, 11% of men and 9% of women remained perceived work ability under the score of 8 (Table 2). Perceived work ability was the best among participants whose work remained light, at 8.8 (CI 8.7–8.9) among both men and women at the end of follow-up.

**Table 2:** Perceived work ability (PWA) (0–10; 0–7 = poor, 8–10 = good) among men and women according to course of workload during 15-year follow-up

Physical strenuousness from 31 years to 46 years								
	Men				Women			
	Always strenuous	Has become more strenuous	Has become lighter	Always light	Always strenuous	Has become more strenuous	Has become lighter	Always light
PWA at 46 years								
Mean	8.3	8.5	8.6	8.8	8.4	8.6	8.7	8.8
(95% CI)	(8.2–8.4)	(8.4–8.7)	(8.4–8.8)	(8.7–8.9)	(8.2–8.5)	(8.4–8.8)	(8.5–8.9)	(8.7–8.9)
0–7, *n* (%)	103 (20)	33 (15)	23 (15)	55 (11)	105 (19)	28 (14)	25 (13)	46 (9)
8–10, *n* (%)	424 (80)	189 (85)	132 (85)	462 (89)	440 (81)	172 (86)	168 (87)	455 (91)

### Association between personal meaning of work and perceived work ability among participants with physically strenuous and light work

The participants who reported low personal meaning of work, both in the strenuous and light work groups, were at approximately a twofold risk (strenuous work; 1.96, 1.51–2.54 vs. light work; 2.11, 1.51–2.95) of poor perceived work ability at 46 years compared to the participants who reported good personal meaning of work (Table 3). The association between personal meaning of work and poor perceived work ability was significant, even after adjusting for the number of diseases, health habits, job strain, social support at work and employment history. Most of these covariates were also independently related to poor perceived work ability. Reporting two or more diseases, unhealthy habits and low social support at work were associated with poor perceived work ability in both the strenuous work and light work groups. Psychological strain was associated with poor perceived work ability in the light work group but not in the strenuous work group. With the exception of poor health habits, the relative risk estimates of the other covariates were lower in strenuous work group than in the light work group (Table 3).

**Table 3:** Association of personal meaning of work (PMOW) and potential covariates for having poor perceived work ability (PWA 0–7) at the age of 46 among participants in strenuous and light work

	Physically strenuous work					
	Yes			No		
	PWA 0–7/n	RR	95% CI	PWA 0–7/n	RR	95% CI
PMOW						
Low	212/896	*1.96*	*1.51–2.54*	139/729	*2.11*	*1.51–2.95*
Medium	95/703	1.21	0.91–1.61	57/676	1.16	0.80–1.68
High	75/709	1.00	–	47/707	1.00	–
Covariates						
Number of diseases						
> 2	344/1460	*2.49*	*1.81–3.43*	226/1637	*3.64*	*2.25–5.89*
0–1	38/504	1.00	–	17/475	1.00	–
Health habits						
Unhealthy	187/903	*1.60*	*1.28–1.99*	90/646	*1.30*	*1.00–1.70*
Between	92/562	1.29	*1.00–1.66*	69/593	1.14	0.85–1.52
Healthy	103/843	1.00	–	84/873	1.00	–
Job strain						
High strain	77/438	1.19	0.89–1.60	64/373	*1.96*	*1.36–2.84*
Low strain	65/433	0.95	0.70–1.29	37/454	1.07	0.71–1.62
Passive	167/870	1.08	0.83–1.41	96/599	*1.62*	*1.14–2.31*
Active	73/567	1.00	–	46/686	1.00	–
Social support at work						
Little	148/680	*1.36*	*1.07–1.72*	105/576	*1.56*	*1.15–2.12*
Somewhat	136/835	1.11	0.88–1.42	81/811	1.02	0.74–1.40
A great deal	98/793	1.00	–	57/725	1.00	–
Employment history						
At least temporary unemployed	194/1082	1.02	0.85–1.22	101/751	1.10	0.87–1.39
Continuously employed	188/1226	1.00	–	142/1361	1.00	–

Multivariate logistic regression analyses adjusted for gender and all other variables in the table, risk ratios (RR) and 95% confidence intervals (CI)

Italics show those RR values, which are statistically significant

## Discussion

This study involving a large population-based birth cohort and covering men and women in all occupational groups and branches of economy showed that perceived work ability decreased from the age of 31 to 46 in both physically strenuous and light work, and was the lowest among participants with physically strenuous work. The participants whose work remained physically strenuous during the 15-year follow-up period perceived their work ability as the worst. Those for whom work had a low personal meaning were at approximately a twofold risk of having poor perceived work ability at 46 years compared to those who reported that work has a high personal meaning to them, in both physically strenuous and light work groups.

### The course of work ability from 31 to 46 years

The findings of Ilmarinen et al. (1997) are in line with the findings of the present study. In that 11-year follow-up study, perceived work ability of aging municipal workers decreased more among those doing physical work compared those doing mental work. The proportion of poor perceived work ability among 31-year-old men with physically strenuous work in this study was about the same level as that reported among 35-year-old operative firefighters (11% and 17%, respectively) (Punakallio et al. 2014). The participants whose work remained strenuous during the 15-year follow-up period reported their work ability to be over 8 (scale of 1–10, 8.3 among men and 8.4 among women), which was a slightly better rating than that among firefighters aged 40–49, who reported work ability of an average of 7.4 (Lusa-Moser at al. 1997). In the strenuous work group, perceived work ability remained always poor or diminished in 22% of men, which is almost the same trend as that found among firefighters, of whom 24% belonged to the declining work ability trajectory during the 13-year follow-up (Punakallio et al. 2014).

We would like to note that despite generally declining perceived work ability over 15 years, most participants, irrespective of the physical strenuousness of their work, perceived their work ability as good. This is also in line with studies of municipal workers, managers and firefighters, of which—despite an overall declining trend—the majority belonged to favorable work ability trajectories (Feldt et al. 2009; Ilmarinen et al. 1997; Punakallio et al. 2014; von Bonsdorff et al. 2011). The high, diverse work demands of physically strenuous work require workers to have good work ability. In the future, more intensive interventions are needed especially among those with decreased work ability already at their early thirties.

### Factors associated with work ability

Low personal meaning of work was significantly associated with poor perceived work ability in both the strenuous work and light work groups, despite adjusting for confounders (number of diseases, health habits, job strain, social support at work, and employment history). We found no earlier studies on the relation between personal meaning of work and work ability among middle-aged workers. Earlier, a more task-related meaning of work, indicating that the work tasks are experienced as meaningful and the results of work as important and useful for others (Kristensen et al. 2005), has been studied as a possible predictor of work absences among eldercare workers (Nielsen et al. 2002, 2004; Tufte et al. 2012), with no clear relation between the experienced meaningfulness of the current work and work absence. Instead, work engagement, another positive work-related motivational concept, has been found to associate with work ability among Finnish firefighters (Airila et al. 2012). Airila et al. (2012) found that during a 10-year follow-up, work engagement was significantly associated with work ability, even after adjusting for several individual and work-related factors such as physical workload. Our results add to the earlier evidence by suggesting that also a more generic positive personal meaning of work seems to have a positive influence on work ability.

Of the other potential predictors, two or more diseases, unhealthy habits and low social support at work were independently related to poor perceived work ability. This accords with the previous findings among workers in physically demanding jobs that many diseases, unhealthy lifestyle habits and poor relationships between workers and supervisors were risk factors for diminished work ability (Lusa et al. 2011; Airila et al. 2012; Punakallio et al. 2014). In this study, a psychosocially high strain job was associated with poor work ability among participants in the light work group, but not in the strenuous work group. This may be because work in the light work group is possibly mentally more demanding than that in the strenuous work group.

With the exception of poor health habits, the risk estimates for the covariates, especially for the number of diseases, were lower in the strenuous work group than in the light work group. One reason for this minor significance of diseases in the strenuous work group compared to the light work group may be that the workers with many diseases had already been excluded (from work and the data). It may be impossible to manage physical tasks with two or more diseases. Previously, both a higher number of comorbid diseases and a high physical workload were shown to be risk factors for disability pension among firefighters (Punakallio et al. 2014). We were somewhat surprised that psychosocial stress factors were associated with perceived work ability in only the light work group, although earlier findings on white-collar workers have shown them to be major factors affecting work ability (e.g. van den Berg et al. 2008).

In an earlier cross-sectional study with different occupations, van den Berg et al. (2011) studied the importance of job control for workers with decreased work ability to remain productive at work. They found that high physical work demands appeared less important for productivity loss at work than psychosocial work characteristics. Alavinia et al. (2009) and Martimo et al. (2009) found similar results. Van den Berg at al. (2011) concluded that job control and related possibilities to adjust work could act as a buffer in highly physically demanding jobs. Nevertheless, in this study, the importance of high personal meaning of work for good work ability was evident, regardless of the level of physical strenuousness of the work.

### Study strengths and limitations

The major strengths of this study included the prospectively collected data and the long follow-up time of 15 years. The data were obtained from a large birth cohort and covered all branches of occupations and sectors of the economy. The men and women were combined and analyses were adjusted for gender. The prospective study setting allowed us to investigate the long-term course of work. However, a loss of participants occurred, as only those living in northern Finland and the metropolitan area were invited to reply to the work-related questions at 31 years. Although the participation rates were high at both 31 and 46 years, the sample size of those who participated in both measurements was smaller. Thus, associations between personal meaning of work and work ability were calculated using a cross-sectional design at the age of 46 only. Although we could adjust our analyses for several covariates, unmeasured residual confounding as well as common method bias always remain possible in an observational study like ours.

Some selection bias occurred, as those excluded from the analyses significantly more often had decreased perceived work ability, unhealthy habits and tended to more often belong to the strenuous work group than the included participants. Some healthy worker effect also occurred. Because the proportions of those with physically strenuous work and poor perceived work ability were higher among the drop-outs, this bias probably somewhat attenuated the observed associations.

### Practical implications

These results have several implications for the promotion of work ability at workplaces, in occupational health care and in society. First, the experience of personal meaning of work appears to be important for work ability in all work. Evaluating the practical possibilities for and methods of externally enhancing an individual’s internally perceived personal meaning of work is beyond the scope of this study, but given the magnitude of the challenge of extending working careers, further studies on this issue are warranted, using different methods. Personal meaning of work is a generic overall perception of the role of work in one’s life and probably a long-lasting attitude towards work. It is likely that personal meaning of work rather slowly develops during the life course in various social contexts. Earlier results using this same cohort support this assumption by showing that school performance at age 16 years predicted high personal meaning of work at 31 years among both genders (Ek et al. 2005). The perceptions of personal meaning of work could possibly to some extent be enhanced at work by providing feedback on the larger benefits one’s work has on, e.g. clients or society, or by helping workers experience success in regard to the goals that are personally important for them. In any case, our results support the idea of highlighting the individual workers’ personal values regarding work in understanding the development of their work ability. Second, since a fifth of the workforce in physically strenuous work presents a notable decline in their work ability already before the age of 46, with at least 20 years before the current statutory retirement age, it is imperative that the actions of disability prevention are begun early and intensively enough at workplaces and in occupational health care; especially by promoting healthy habits among workers in physically strenuous work.

## Conclusions

Perceived work ability was significantly lower and deteriorated more over 15 years’ follow-up in middle age among the participants with physically strenuous compared to those with light work. Personal meaning of work was important for good work ability in both physically strenuous work and light work, and should, therefore, be taken into account in work ability promotion at workplaces and in occupational health care.
